# Prenatal diagnosis: the clinical usefulness of array comparative genomic hybridization

**DOI:** 10.1016/j.pbj.0000000000000013

**Published:** 2018-07-03

**Authors:** Marta Freitas, Joel Pinto, Carla Ramalho, Sofia Dória

**Affiliations:** a Faculty of Medicine, University of Porto, Portugal; b Genetics Unit, Department of Pathology, Faculty of Medicine; c I3S – Instituto de Investigação e Inovação em Saúde, University of Porto; d Department of Gynecology and Obstetrics, Centro Hospitalar de São João, EPE; e Department of Gynecology, Obstetrics and Pediatrics, Faculty of Medicine, University of Porto, Porto, Portugal.

**Keywords:** array comparative genomic hybridization, copy number variation, karyotype, prenatal diagnosis, variants of unknown significance

## Abstract

**Background:**

Array comparative genomic hybridization (aCGH) has been replacing karyotype in neurodevelopment diseases or intellectual disability cases. Regarding prenatal diagnosis (PND) karyotyping is still the criterion standard technique; nevertheless, the application of aCGH in this field has been increasing dramatically and some groups recommended it as the first-tier prenatal genetic test in cases of fetal ultrasound abnormalities. Despite aCGH greater resolution, the detection of variants of unknown significance (VOUS) is not desirable, so it's need some reflexion before generalized application on PND.

**Objective:**

The aim of this study was to analyze the prevalence and type of copy number variants (CNVs) detected in the 55 PND samples collected from pregnancies with indication to perform aCGH.

**Methods:**

aCGH was performed using Agilent 4 × 180K microarrays and results were analyzed using CytoGenomics software.

**Results and conclusion:**

Eight (14.5%) cases had pathogenic or likely pathogenic CNVs. VOUS were found in 21.8% of the cases, but this frequency could be minimized if only large CNVs above 1 million base pairs that are outside the clinically curated targeted regions were considered.

## Introduction

Array comparative genomic hybridization (aCGH) is a useful technique for the detection of DNA submicroscopic rearrangements, known as copy number variants (CNVs), using molecular technologies. With this technique, both patient and control samples are labeled with 2 different-colored fluorescent dyes combined and hybridize at the array platform. After, the fluorescence intensity ratio is measured and areas that are under-represented or over-represented are quantified.^1,2^

The application of aCGH on routine chromosomal analysis has substantially increased the diagnostic yield in clinical cytogenetics and nowadays it is assumed to be the first genetic test used for postnatal diagnosis in cases of intellectual disability or neurodevelopment diseases. However, regarding prenatal diagnosis, karyotyping, which has a resolution of 5 to 10 Mb, is still the criterion standard technique.^2–4^ The greater resolution of aCGH, about 100 times higher than karyotype, enables higher detection rates of CNVs and also the detection of microscopic imbalances that are seen as balanced chromosomal translocations in karyotyping, which occurs mainly when caused by a genomic gain or loss at the translocations breakpoints.^1^ The possibility of replacing karyotyping by aCGH or by aCGH in addition to multiplex ligation-dependent probe amplification (MLPA) or quantitative fluorescence-polymerase chain reaction (QF-PCR) has been increasingly discussed among scientific community, namely in fetus with ultrasound abnormalities.^3,5–7^

In 2012, multicenter trial sponsored by the National Institute of Child Health and Human Development (NICHD) showed that in prenatal cases with a normal karyotype, aCGH provided additional relevant information in 6.0% of the cases with an ultrasound anomaly and in 1.7% of those with standard indications for an invasive prenatal test. Furthermore, aCGH analysis was considered equivalent to karyotype concerning the detection of common aneuploidies.^1^ A 2013 systematic review reported that in cases with normal karyotype, aCGH revealed at least 1 CNV clinically significant in 2.4% (295/12362) of overall prenatal cases.^8^

Today both American College of Obstetricians and Gynecologists and Society for Maternal-Fetal Medicine recommend the performance of aCGH (replacing karyotype) as the first-tier genetic testing in cases of fetus with ultrasound abnormalities undergoing invasive prenatal diagnosis.^2,9^

The major concerns are the high cost of aCGH analysis and mainly the detection of variants of unknown significance (VOUS). A VOUS is a copy number variation for which there is none or few data correlating it with a defined clinical phenotype. These findings may present a challenge regarding interpretation and genetic counseling.^7,10^

The purpose of this study was to analyze the prevalence and type of CNVs detected in the 55 samples collected from pregnancies occurred between June 2013 and June 2016 followed in Centro Hospitalar São João (CHSJ) that had the indication to perform prenatal diagnosis using aCGH. Our goal was to correlate the imbalances detected with the phenotype (ultrasound findings) and evaluate the current indications to perform prenatal aCGH in our hospital. Finally, it was also our concern to reflect about the interpretation and the impact on prenatal diagnosis and genetic counseling of the detected VOUS.

## Methods

**Figure 1:**
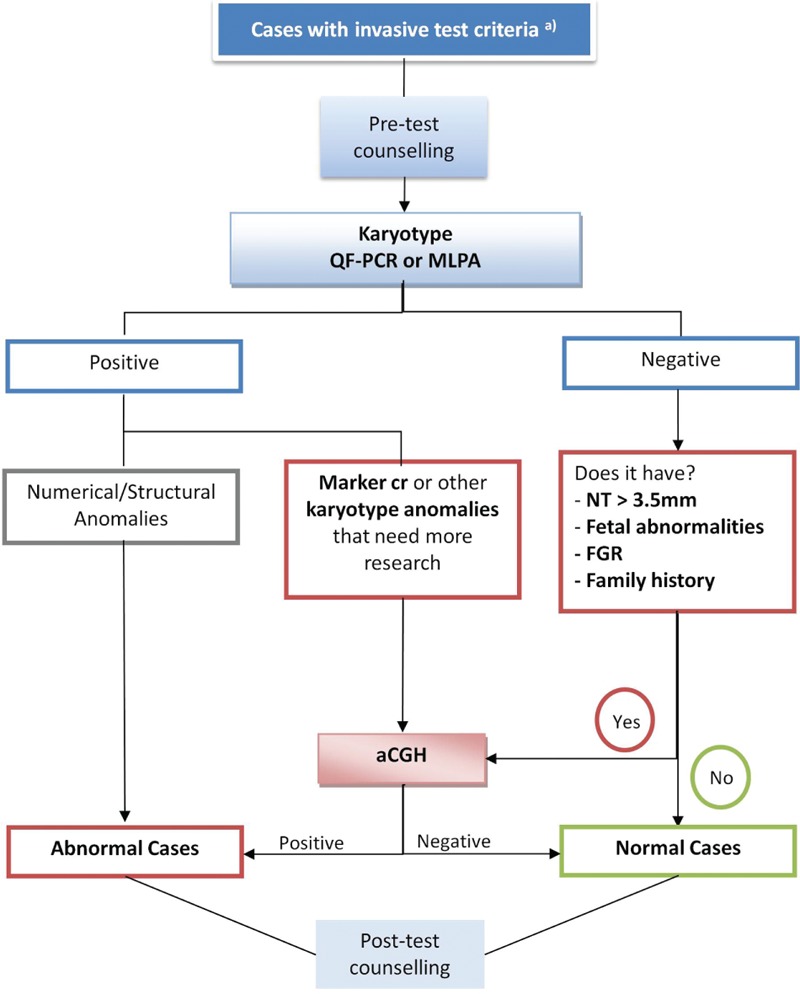
Algorithm of genetic prenatal diagnosis in Centro Hospitalar São João (CHSJ): tests and criteria. ^a^Indications for invasive pregnancy test at CHSJ: positive combined first trimester screening, fetal abnormalities, nuchal translucency (NT) >3.5 mm (P99), early-onset fetal growth restriction (FGR), parents with balanced chromosomal rearrangements, family history of genetic disorder, and maternal anxiety.

Fifty-five prenatal samples were analyzed at the Genetic Unit, Pathologic Department, Oporto Faculty of Medicine, between June 2013 and June 2016. CHSJ in Oporto, Portugal, monitored all patients. All women with indication to perform an invasive prenatal diagnostic test had pretest genetic counseling and signed an informed consent for invasive technique, karyotype, and aCGH. aCGH was carried out if at least one of the following indications were present: nuchal translucency higher than percentile 99 (3.5 mm), fetal abnormalities, early-onset fetal growth restriction (FGR), family history of genetic imbalances, or cases with abnormal karyotype (those in which there was the need of clarifying the result and in cases of de novo balanced rearrangements) (Fig. 1).

From the 55 samples, 50 were singleton pregnancies, 4 from 2 monochorionic diamniotic twin pregnancies and 1 from a dichorionic diamniotic pregnancy (parents decided to test only the fetus with ultrasound anomalies). Samples of amniotic fluid or of chorionic villus were collected in each case, according to the gestational age. In 1 case, it was collected fetal blood during delivery (Table 1).

**Table 1:**
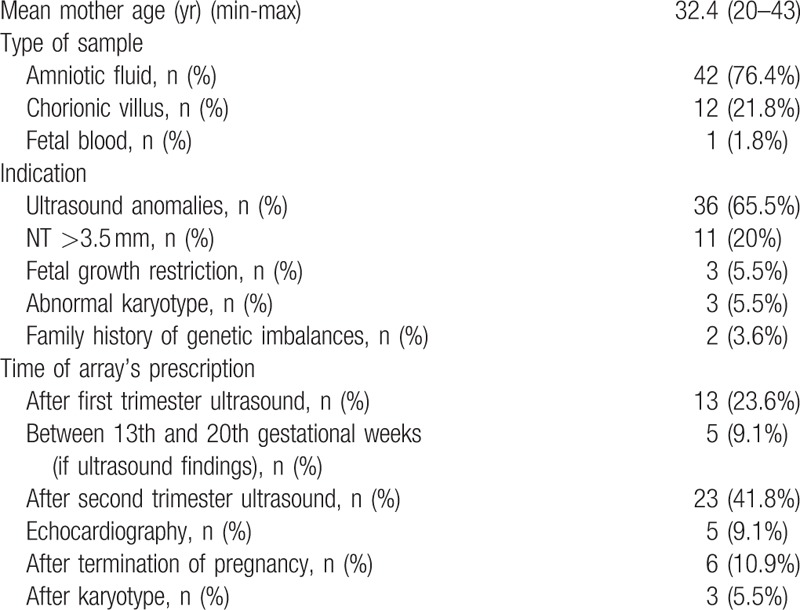
Sample characterization

aCGH was performed using Agilent SurePrint G3 Human Genome 4 × 180K microarrays (Agilent Technologies, Santa Clara, CA). These microarrays contain approximately 180,000 probes with a 13 kb average probe spacing. Results were analyzed using CytoGenomics software. In order to decrease the VOUS detection rate, the software settings were adjusted, so that only CNVs >200 kb and that included at least 5 consecutive probes with abnormal log_2_ ratios, could be detected. There is no consensus at European level about the resolution that should be used. Most European countries guidelines recommend the use of CGH array platforms for prenatal diagnosis with a total minimum resolution of about 400 kb.^11^ Belgian guidelines recommend the use of a 60K or a similar array platform.^12^ We decide to follow these guidelines.

For interpretation of the results we used the following databases: DGV, OMIM, DECIPHER, and CLINGEN. CNVs were classified as pathogenic, benign, VOUS and VOUS likely pathogenic or likely benign. This classification is in accordance with the American College of Medical Genetics Standards and Guidelines.^13^ The genetic study of the progenitors was performed only in the cases with pathogenic or likely pathogenic CNVs, due to Hospital financial restrictions. Just in one of the cases with VOUS was decided to perform progenitors’ studies, because there was a family history of an autism spectrum disorder. This study was done using MLPA.

The statistical analysis in this work was performed using IBM SPSS Statistics 24.0.

The CHSJ Ethical Committee approved this study.

## Results

Pathogenic or likely pathogenic CNVs were detected in 8 of the 55 cases (14.5%), 5 of those 8 cases (9.1%) had a normal karyotype (Table 2). VOUS were found in 12 cases (21.8%). A table with complete information of all VOUS and VOUS likely benign found is provided as supplementary material. In 18 (32.7%) the progenitors decided to perform termination of pregnancy (TOP) and in 1 case occurred a fetal death on the second trimester. In the other 36 cases, the pregnancy continued until delivery, with a neonatal death. The cases classified as pathogenic or likely pathogenic are the following (Table 2).

**Table 2:**
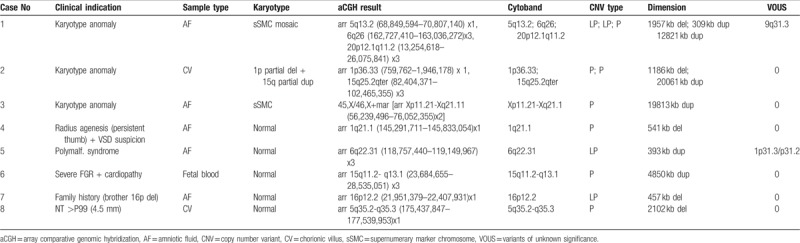
aCGH cases with pathogenic or likely pathogenic CNVs

### Case 1

A chorionic villus sample (CVS) was collected at 13 weeks gestation because of a positive combined test (risk 1:300). Karyotype revealed the presence of a mosaic supernumerary marker chromosome (sSMC) (47,XY,+mar[7]/46,XY[15]). In order to confirm and identify the origin of the sSMC, an amniocentesis was done to perform aCGH in the amniotic fluid sample. The analysis showed 2 likely pathogenic CNVs and a pathogenic CNV (case 1, Table 2). The 5q13.2 deletion (1957 kb) contains genes associated with spinal muscular atrophy and the 6q26 duplication (309 kb) has been associated with neurological alterations, micrognathia, and developmental delay.^14,15^ Nevertheless, none of these variations should relate with the presence of the marker, but they could also be pathogenic. The extension of the 20p12.1q11.2 duplication, about 12.8 Mb, explains all by itself the presence of sSMC and allows his identification as material from chromosome 20. The higher and relevant gene content (85 genes) suggest a pathogenic contribution. A fluorescence in situ hybridization test using a centromeric probe for chromosome 20 confirmed the aCGH result, establishing a degree of mosaicism of approximately 35%. Marker chromosomes are associated with high phenotypic variability, especially the ones involving chromosome 20.^16^ Furthermore, this alteration is not present at 100% of the cells, the phenotypic consequences of this duplication are difficult to predict. However, for the reasons explained above, a high risk of association to pathology for this gestation is considered. Progenitors decided for TOP and the necropsy examination detected a few *minor* development anomalies, without *major* anomalies.

### Case 2

The motive for CVS at 13-week gestation was a positive combined test (risk of 1:9 for trisomy 21 and 1:19 for trisomy 13). An abnormal chromosome 1 with additional material attached on the p arm was observed on the karyotype suggesting a derivative chromosome. The parents’ karyotypes were normal so the karyotype was defined as 46,XX,add(1)(p36.1) de novo. An aCGH was carried out to clarify this finding and found 2 pathological CNVs: a 1p36.33 deletion (1186 kb, including 55 genes) and a 15q25.2qter duplication ((20061 kb, containing 144 genes). Both associated to severe phenotypic alterations such as hypotonia, developmental and/or mental retardation, cardiac, and renal malformations.^17,18^

Progenitors decided for TOP and the necropsy examination showed a female fetus with *minor* development anomalies consistent with the diagnosis of structural chromosomal abnormality, without *major* anomalies.

### Case 3

Amniocentesis was performed at 16-week gestation because of a positive combined test (with increased risk for trisomy 18). The karyotype revealed mosaicism, a 45,X cell line (the most predominant) and a cell line with 46 chromosomes showing a marker chromosome (45,X[22]/46,X,+mar[8]). Even though the cell line with the marker was minority, in approximately 36% of the cells, aCGH technique was able to identify the origin of the marker chromosome, showing Xp11.21-Xq21.1 duplication of 19813 kb (because it was hybridized with a male control). This CNV is a pathogenic one associated to a variant of Turner syndrome.^19,20^

Progenitors decided to continue the pregnancy and an FGR diagnosed in the third trimester. At 37 weeks the newborn weighted 2309 g and had no other apparent malformations.

### Case 4

Fetal anomalies (radius agenesis with persistent thumb and suspicion of ventricular septal defect) diagnosed at 22-week gestation were the reason to perform amniocentesis. Karyotype was apparently normal, but a 541 kb deletion on 1q21.1 was detected on aCGH. This deletion includes the *RBM8A* gene, which is associated with the thrombocytopenia absent radius (TAR) syndrome.^21^ Nevertheless, once it is an autosomal recessive disease an additional mutation in the other allele should be found.

Even before the aCGH result, progenitors decided for TOP and the necropsy examination showed a female fetus with anomalies compatible with TAR syndrome: right radius agenesis and severe left radius hypoplasia with persistent thumb (bilaterally).

### Case 5

Amniocentesis performed at 21-weeks’ gestation because of multiple fetal abnormalities (strawberry-shaped skull, enlarged orbits, right congenital diaphragmatic hernia, and ventricular septal defect). The aCGH detected a 6q22.31 duplication of 393 kb, which contains the *PLN* gene. According to the literature this gene has a relevant function in contractility and relaxation of the cardiac cells and mutations in this gene are associated to cardiomyopathy.^22,23^

Nevertheless, it's not clear if a duplication can produce the same effect of a mutation (just 1 case of a duplication causing dilated cardiomyopathy was describe in literature), so this CNV was classified as likely pathogenic.^24^

TOP was done before the aCGH result. The necropsy examination showed congenital defect of the diaphragm, upper and lower limbs anomalies, decreased bone density in long bones, and liver with anomalous morphology.

### Case 6

A severe FGR and tetralogy of Fallot detected at 31 weeks. During cesarean section fetal blood from umbilical cord was collected for genetic analysis. Growth restriction (weight of 1035 g at 33 weeks) and tetralogy of Fallot confirmed in the newborn.

The aCGH revealed a duplication on 15q11.2-q13.1 of 4850 kb, which includes 101 genes that overlaps, although not in the entire genomic range, with the 15q11.2-q13.1 microduplication syndrome. This syndrome is associated with developmental and intellectual delay, autism, microcephaly, face malformations, prominent ears, among other anomalies.^25^ There are several cases describing this alteration as an inherit CNV, especially from maternal origin with a variable penetrance.^26^ MLPA test for this specific duplication was done on the mother, revealing a normal result, confirming that it is not inherited from the mother. Father was not available for this study.

### Case 7

A couple with a previous child with intellectual disability, left-sided hemiparesis, cystic hepatic lesions, and multiple skin hemangiomas followed in the genetic consultation. The aCGH performed in the child found a 457 kb microdeletion syndrome on 16p12.2. The mother is carrier of the same deletion with normal phenotype. In the present pregnancy, amniocentesis performed at 18-weeks’ gestation and aCGH revealed the same deletion present in the mother and brother. This 16p12.2 microdeletion is included in the 16p12.2-p11.2 deletion syndrome. It is characterized by developmental delay, cognitive and growth impairment, cardiac malformations, epilepsy, autism, psychiatric and/or behavioral problems, and other congenital malformations.^27,28^ According to the literature there are several phenotypes described for this region, being deletions located more centromeric (in 16p11.2) more prone to be associated with autism.^27–29^ In our patient the deletion affects 16p12.2 region. This region is distinct from p11.2 region and is involved in multiple congenital anomalies and intellectual disability phenotype.^28^ In the database DECIPHER, several patients with similar deletions affecting 16p12.2 regions (408–494 kb) showed commonly intellectual disability. Other features were autism behavior, kidney and limb abnormalities, and other variable malformations. The majority of affected individuals described have inherited the microdeletion from a parent who can or cannot have clinical features caused by the microdeletion. According to Girirajan et al^29^ patients with the microdeletion were found to be more likely to have clinical findings such as seizures, mild intellectual disability, and/or psychiatric issues; suggesting that the 16p12.2 microdeletion is a risk factor for abnormal neurodevelopmental phenotypes with reduced penetrance and variable expressivity.^29^

Progenitors decided for TOP and the necropsy examination identified in the fetus some limb and craniofacial anomalies (low insertion of the auricles, upper jaw hypoplasia), without apparently other *major* anomalies.

### Case 8

CVS was collected at 14-weeks’ gestation because of nuchal translucency of 4.5 mm. The aCGH revealed a 2102 kb 5q35.2-q35.3 deletion involving several genes including *NSD1* gene, which is associated with Sotos syndrome.^30^ This syndrome has been described in several patients with mental retardation, cardiac anomalies, renal anomalies, scoliosis, and seizures.^31^ MLPA test for this region was done on both parents and the result was normal, suggesting a de novo deletion.

Progenitors decided to continue the pregnancy and an Ebstein anomaly diagnosed in the newborn (weight of 3520 g at 40 weeks).

## Discussion

According to our results, aCGH can be a very useful test in prenatal diagnosis. In 9.1% of the cases no anomaly would be detected in the prenatal period if aCGH was not performed, once karyotype was normal. In cases 1, 2, and 3 aCGH also contributed to a better interpretation of the anomalies found in karyotype improving the post-test genetic counseling. According to literature, aCGH can provide additional diagnostic information over karyotype in 5.1%^3^ to 8.2%^32^ of the cases in prenatal diagnose (6.0% in NICHD study).^8^ In the present study an abnormal result was obtained in 9.1% of the cases with normal karyotype. This higher detection rate could be explained by the specific indications included in this study (nuchal translucency higher than percentile 99, fetal abnormalities, early-onset FGR, and family history of a genetic imbalance). In addition, it should be stress out that cases classified as likely pathogenic were also included in this rate detection (Table 2). If we consider only pathogenic cases and exclude cases with abnormalities in the karyotype, our detection rate is 5.45% (3/55). When we include all the cases, the detection rate is 10.9% (6/55). These rates are similar to the literature.

However, the high rate detection of VOUS remains a challenge to prenatal diagnosis and genetic counseling. VOUS can be minimized by reporting only large CNVs that are outside clinically curated targeted regions. Some authors only report CNVs of 1 million base pairs or larger.^10^ With the increasing experience, one expect that many previously uncertain findings to be reclassified as benign or pathogenic as additional information is gathered in publically shared clinical databases.^10^ The access to regularly updated databases, which help the correlation genotype-phenotype for CNV in the postnatal setting, will provide essential interpretative tools in the prenatal diagnosis.^8^ The adjustment of software analysis parameters is also a solution pointed out, to reduce VOUS rate without missing any relevant CNV.^33^

In our study VOUS were found in 22% of the cases. This high rate of detection could be explained because parental studies, using MLPA or QF-PCR, were only performed for pathogenic or likely pathogenic CNVs. VOUS or VOUS likely pathogenic were not systematically tested in the progenitors, due to hospital financial restrictions. Progenitor's study, using MLPA or QF-PCR, is important for pathogenic or likely pathogenic CNVs and should be considered for VOUS or VOUS likely benign regarding the clinical context, to confirm if it is an inherit or a de novo imbalance. Inherit variants are considered of lower risk in comparison with de novo variants. Nonetheless, inheritance cannot be used as a sole parameter of genotype-phenotype correlation, this means that we cannot say that an inherit CNV is always benign, once some CNVs have a variable outcome.^2^ This information is extremely useful in the interpretation of the CNVs detected and it's also very relevant in genetic post-test counseling. This study allowed us to address the importance of performing, in our hospital, parental studies for all CNVs (not classified as benign) found in the fetus.

Another main concern regarding aCGH analysis is the possibility of detecting CNVs associated with adult-onset disorders (such as *BRCA* mutations, Charcot-Marie-Tooth disease). This is a problem not only regarding the fetus (which information should be given in post-test counseling), but also related to the progenitors, because the CNV detected can be inherited from an asymptomatic parent and represent risk for a future disease to him. It is crucial to discuss how to manage these situations, to inform the progenitors, and make them understand the possible results of aCGH analysis. Therefore, a protocol concerning the aCGH best practices should be included in clinical practice. At the moment, no current European guideline has yet been published. Nevertheless, Cytogenomic External Quality Assessment Service is now preparing a document concerning the best practices in prenatal array, based on the consensus of the most experienced European Laboratories. It is expected that this could be the next European guidelines for the use of arrays in prenatal diagnosis.

Regarding the usefulness of aCGH in prenatal diagnosis it is of value to analyze the classical limitations pointed out to this technique, such us the detection of polyploidy, balanced chromosomal rearrangements, inversions, and low mosaicism.^2,10^ Moreover, aCGH does not provide information about the chromosomal mechanism of the genetic imbalance, which could be important to inform about future recurrence risk.^2^ However, some changes can be made to circumvent these limitations, for example, the incorporation of single nucleotide polymorphisms probes into aCGH allows the detection of polyploidy.^10^ Regarding balance rearrangements, it is known that it occur in approximately 0.08% to 0.09% of the prenatal samples and most of them result in a normal outcome (mostly, the concern is to future reproductive counseling).^1,2^ A de novo, apparently balanced rearrangement detected in karyotyping is associated with a 6.7% risk of congenital abnormalities, mainly because of a genomic gain or loss at the breakpoints that cannot be seen with karyotyping.^1^ These de novo balanced translocations can have cryptic intrachromosomal rearrangements in addition to the cytogenetically visible structural chromosome alteration. Therefore, aCGH analysis could be very important, not only to detect genomic imbalances at the breakpoints, but also to detect unexpected rearrangements in other chromosomes.^34^ Another concern with aCGH is that it cannot detect low-level mosaicism (<10%–20%), but mosaicisms <14% could also escape to the karyotype observation (it only detects mosaicism of 14% with 95% confidence). It is worth mentioning that this low-grade mosaicism frequently has no relevant phenotype associated.^10,35^ Furthermore, aCGH does not require culture cells, so DNA extracted directly from uncultured cells can reveal mosaicism that could not be visible in cultured cells because of the preferential growth of a normal cell line.^10^ It should be noted that comparing to karyotype aCGH has similar specificity (99% vs 98.7%), but higher sensitivity (67.3% vs 94.5%).^36^ Another issue is the aCGH price, which is much more expensive than karyotyping. However, the studies conducted to evaluate the cost effectiveness of these techniques, conclude that performing aCGH alone is the most cost-effective strategy in the prenatal diagnosis of detected ultrasound fetal anomalies.^37^

For all of these reasons aCGH is a technique that add a great value in the prenatal diagnosis setting and it's expected that in the near future it replaces the karyotype as the first genetic test in this field. In our institution aCGH improved prenatal diagnosis and genetic counseling. The evaluation in terms of cost effectiveness of the possibility of using aCGH test in every case with indication to perform an invasive test or after excluding common aneuploidies by QF-PCR should be performed in our hospital.

## Acknowledgments

None.

## Conflicts of interest

The authors declare that they have no competing interests.
